# Construction of autophagy prognostic signature and analysis of prospective molecular mechanisms in skin cutaneous melanoma patients

**DOI:** 10.1097/MD.0000000000026219

**Published:** 2021-06-04

**Authors:** Shian Liao, Juliang He, Chong Liu, Zide Zhang, Hongyu Liao, Zuowei Liao, Chaojie Yu, Jian Guan, Hao Mo, Zhenchao Yuan, Tuo Liang, Zhaojun Lu, Guoyong Xu, Zequn Wang, Jiarui Chen, Jie Jiang, Xinli Zhan

**Affiliations:** a Spine and Osteopathy Ward, The First Affiliated Hospital of Guangxi Medical University, Nanning, Guangxi; b Department of Bone and Soft Tissue Surgery, Guangxi Medical University Cancer Hospital; c Guangxi Medical University, Nanning, Guangxi; d Southern Medical University, Guangzhou, Guangdong; e Department of General Surgery, The Ninth Affiliated Hospital of Guangxi Medical University, Beihai, Guangxi, China.

**Keywords:** autophagy prognostic signature, IHC, molecular mechanisms, nomogram, SKCM

## Abstract

**Background:**

Autophagy is closely related to skin cutaneous melanoma (SKCM), but the mechanism involved is unclear. Therefore, exploration of the role of autophagy-related genes (ARGs) in SKCM is necessary.

**Materials and methods:**

Differential expression autophagy-related genes (DEARGs) were first analysed. Univariate and multivariate Cox regression analyses were used to evaluate the expression of DEARGs and prognosis of SKCM. Further, the expression levels of prognosis-related DEARGs were verified by immunohistochemical (IHC) staining. Finally, gene set enrichment analysis (GSEA) was used to explore the underlying molecular mechanisms of SKCM.

**Results:**

Five ARGs (*APOL1*, *BIRC5, EGFR, TP63,* and *SPNS1*) were positively correlated with the prognosis of SKCM. IHC verified the results of the differential expression of these 5 ARGs in the bioinformatics analysis. According to the receiver operating characteristic curve, the signature had a good performance at predicting overall survival in SKCM. The signature could classify SKCM patients into high-risk or low-risk groups according to distinct overall survival. The nomogram confirmed that the risk score has a particularly large impact on the prognosis of SKCM. Calibration plot displayed excellent agreement between nomogram predictions and actual observations. Principal component analysis indicated that patients in the high-risk group could be distinguished from those in low-risk group. Results of GSEA indicated that the low-risk group is enriched with aggressiveness-related pathways such as phosphatidylinositol-3-kinase/protein kinase B and mitogen-activated protein kinase signalling pathways.

**Conclusion:**

Our study identified a 5-gene signature. It revealed the mechanisms of autophagy that lead to the progression of SKCM and established a prognostic nomogram that can predict overall survival of patients with SKCM. The findings of this study provide novel insights into the relationship between ARGs and prognosis of SKCM.

## 1 Introduction

Skin cutaneous melanoma (SKCM) is a malignant tumour caused by the malignant transformation of melanocytes.^[1,2]^ The morbidity and mortality rates due to SKCM have been increasing since the last half-century.^[3]^ Globally, approximately 2,30,000 (1.7%) of all newly diagnosed primary malignant tumours (excluding non-SKCM skin cancer) constitute SKCM, and nearly 60,000 SKCM patients die every year (accounting for 0.7% of all cancer deaths).^[4]^ SKCM progresses rapidly, and once it is metastasised to other organs, it cannot be treated. Because of lack of effective treatment options for such cases, patients’ life expectancy is up to 2 years. Early diagnosis and treatment are the keys to improve the prognosis and survival time in patients with SKCM. Although the SKCM diagnosis and treatment criteria established by the American Joint Cancer Council provide SKCM staging and guidance for the treatment, they cannot be used to determine the risk of early tumour progression and to obtain reliable stratification for new adjuvant therapies.^[5]^

Autophagy is a primary catabolic process in which the cells remove damaged, defective, or useless organelles, long-lived proteins, and lipids in the cytoplasm and recover their components to meet their nutritional and energy requirements for biological metabolism.^[6]^ Since the elucidation of the mechanism of autophagy by a Japanese researcher Yoshitoku Oi, who won the 2016 Nobel Prize in Physiology or Medicine, the role of autophagy in clinical medicine has been well-emphasised. Increasing studies have shown that autophagy plays an essential role in the development of cancer. It is noteworthy that autophagy plays different roles in cancer. Autophagy is believed to inhibit the occurrence of cancer; however, on the other hand, once the cancer is established, the increase in autophagy flux can also promote cancer development.^[6,7]^ Evidence suggests that autophagy can promote invasion and metastasis in case of SKCM.^[8]^ Xie et al^[9]^ reported that autophagy-related gene-7 promotes SKCM by limiting oxidative stress and overcoming aging and that autophagy inhibition may enhance the anti-tumour activity of BRAF inhibitors, and thus may have therapeutic value. Therefore, if certain autophagy-related genes (ARGs) can be used to construct models and accurately determine prognosis in patients with SKCM, it will help in better risk assessment and in improving the personalised management of SKCM patients in the clinic. The expression of differentially expressed autophagy-related genes (DEARGs) plays a key role in the development of SKCM, and it can be an independent biomarker for predicting the prognosis of SKCM.^[10]^ The function of autophagy in SKCM is complex, and in-depth understanding of the role of autophagy in the occurrence and development of SKCM is lacking. Therefore, investigation of the relationship between autophagy and SKCM might provide a new reference index for the stratification of prognosis risk and selection of an appropriate treatment strategy for patients with SKCM. Therefore, in this study, we aimed to combine bioinformatics with basic experiments to discover the relationship between ARGs and prognosis of SKCM and its molecular mechanism and to construct a model that can accurately predict the prognosis of SKCM.

## 2 Materials and methods

### 2.1 Gene expression datasets and ARGs

ARGs were obtained from The Human Autophagy Database (HADb, http://www.autophagy.lu/index.html). Gene expression data of 471 patients with SKCM of HTSeq-FPKM type and corresponding clinical data and gene expression data used to match the normal skin of SKCM were obtained from the University of California, Santa Cruz Xena (UCSC, Xena; http://xena.ucsc.edu/).

### 2.2 Functional enrichment analysis of ARGs

To learn more about the functions of ARGs, the cluster Profiler package in R (http://cran.r-project.org/; version 3.6.3) was used to perform Gene Ontology (GO) and Kyoto Encyclopedia of Genes and Genomes (KEGG) pathway analyses on these genes.

### 2.3 Screening of DEARGs

The limma package in R was used to screen DEARGs. The log2|fold change| ≥ 2.5 and false discovery rates (FDR) <0.05 were considered as the cut-off criteria for DEARGs.

### 2.4 Construction of the autophagy prognostic signature

Univariate Cox regression analyses were performed to select the ARGs whose expression profiles are significantly associated with OS of patients with SKCM. Subsequently, these patients’ survival-related genes were subjected to a multivariate Cox regression analysis to identify highly prognostically correlated genes and construct a autophagy prognostic signature. To evaluate the autophagy prognostic signature's predictive value, a time-dependent receiver operating characteristic (ROC) curve was constructed using the survival ROC package. Survival analysis was performed to compare the prognosis between high-risk and low-risk groups.

### 2.5 Immunohistochemical staining

The SKCM group comprised 10 patients with SKCM who visited the First Affiliated Hospital of Guangxi Medical University between January 2018 and August 2020. Patients diagnosed as having cutaneous melanoma through surgery for the first time and in the routine pathological examination after surgery in our hospital were included. Exclusion criteria were as follows: (1) patients who had been operated in other hospitals and presented to our hospital for extended resection; (2) patients with other types of tumor; (3) patients with mucosal melanoma in the eyes, mouth, nose, anus, and genitalia; (4) patients having received radiotherapy, chemotherapy, or immunotherapy before surgery; and (5) patients with skin diseases, immunodeficiency diseases, diabetes, hypertension, or coronary heart disease. Ten patients with normal skin who underwent plastic surgery in the First Affiliated Hospital of Guangxi Medical University due to trauma were selected as the control group. None of these patients had skin diseases, immunodeficiency diseases, diabetes, hypertension, coronary heart disease, or tumor. This study was approved by the Institutional Review Board of Ethics Committee of The First Affiliated Hospital of Guangxi Medical University (Approval Number: 2021(KY-E-025)). Informed consent was obtained from each patient.

SKCM samples and normal skin samples were preserved in 2.5% glutaraldehyde-polyoxymethylene solution. The samples were then dehydrated and embedded in paraffin following routine methods and processed as 5-μm continuous sections. Each group of sample was dewaxed with discontinuous concentrations of ethanol and blocked to inhibit endogenous peroxidase. Thereafter, the samples were heated in a microwave to retrieve antigens, cooled to room temperature, and blocked by incubation with goat serum (diluted with PBS) for 30 minutes at 37 °C. Samples were incubated overnight with rabbit anti- APOL1 (Bioss Inc., Beijing, China; bs-12498R; 1:500 dilution), rabbit anti-survivin (Bioss Inc., Beijing, China; bs-0615R; 1;600 dilution), rabbit anti-EGFR-specific (Proteintech, Wuhan, China; 18986-1-AP; 1:800 dilution), rabbit anti-P63 (Bioss Inc., Beijing, China; bs-0723R; 1:300 dilution), and anti-SPNS1 (Bioss Inc., Beijing, China; bs-7078R; 1:400 dilution) antibodies at 4 °C, followed by incubation with horseradish peroxidase-coupled goat anti-rabbit secondary antibody at 37 °C for 30 minutes, and staining with 3,3′-diaminobenzidine. Sections were then dehydrated, cleared by xylene, and mounted. Expressions of APOL1, BIRC5, EGFR, TP63, and SPNS1 were detected through the immunohistochemical (IHC) analysis by using the streptavidin–peroxidase technique, according to the instructions.^[11]^

### 2.6 Independent prognostic value of the autophagy prognostic signature

The multivariate Cox regression analysis was conducted to investigate if the autophagy-related risk score could be an independent predictor of OS in patients with SKCM. The risk score, age, gender, and pathological stages (T, M, N) were used as covariates. A *P* value of <.05 was considered significant. Meanwhile, we calculated the hazard ratio (HR) and 95% confidence interval (CI). A nomogram was constructed based on clinical variables and risk scores of autophagy prognostic signature. The scale marked on the line represents the range of values for each variable, and the length of the line segment reflects the contribution of this factor to the outcome event. In addition, the calibration plot was used to verify the accuracy of the nomogram.

### 2.7 Principal components analysis and gene set enrichment analysis

Principal component analysis (PCA) was performed using the “pca3d” package to study gene expression patterns in grouped patients. Gene set enrichment analysis (GSEA) (https://www.gsea-msigdb.org/) was conducted between high-risk and low-risk phenotypes. The enrichment gene sets in the GSEA that attained FDR of <0.25 and *P* value of <.05 were considered significant.

## 3 Results

### 3.1 Functional enrichment analysis of ARGs

**Figure 1:**
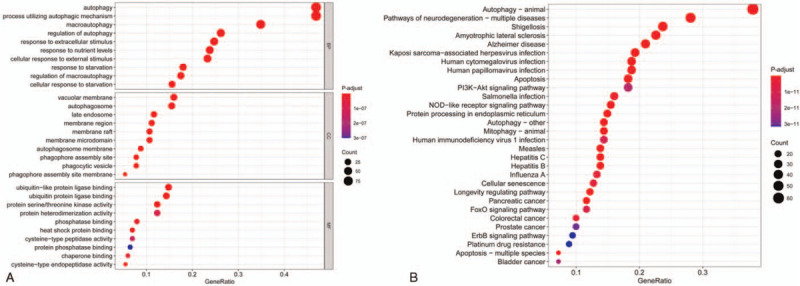
Enrichment analysis of GO terms and KEGG pathways of ARGs. (A) GO terms. (B) KEGG pathways. ARGs = autophagy-related genes, BP = biological process, CC = cellular component, GO = Gene Ontology, KEGG = Kyoto Encyclopedia of Genes and Genomes, MF = molecular function.

As shown in Figure 1, ARGs were found to be mainly enriched in the regulation of autophagy, epithelial cell proliferation, T cell proliferation, B cell activation, and phosphatidylinositol-3-kinase/protein kinase B (PI3K-Akt) and mitogen-activated protein kinase (MAPK) signalling pathways in GO terms and KEGG pathways.

### 3.2 Identification of DEARGs in SKCM

**Figure 2:**
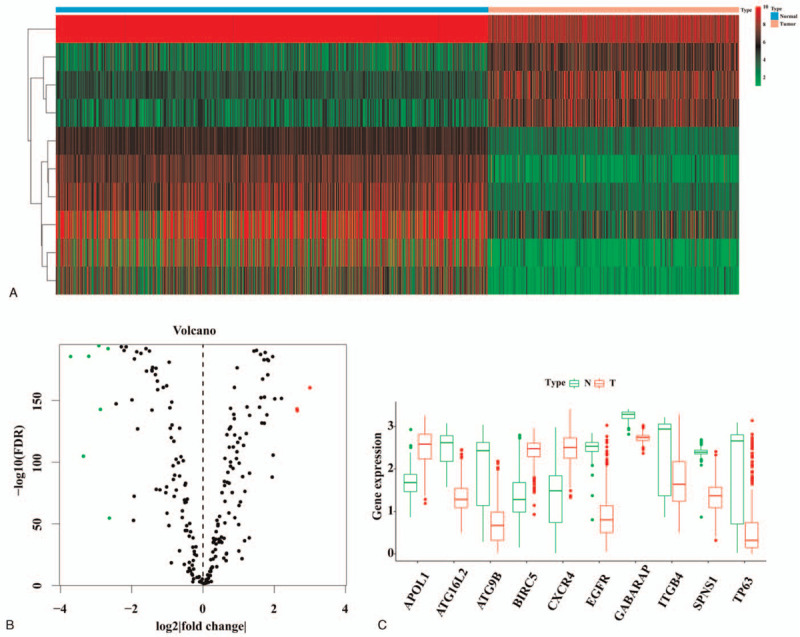
Identification of DEARGs in SKCM. (A) Heatmap of DEARGs. (B) Volcano map of DEARGs. (C) Boxplots displayed expression patterns of 7 downregulated and 3 upregulated DEARGs. DEARGs = differentially expressed autophagy-related genes, FDR = false discovery rate, SKCM = skin cutaneous melanoma.

Through careful screening, we finally identified 3 significantly upregulated and 7 significantly downregulated DEARGs. Boxplots displayed expression patterns of 7 downregulated (*EGFR, GABARAP, TP63, ITGB4, ATG9B, SPNS1,* and *ATG16L2*) and 3 upregulated DEARGs (*APOL1, BIRC5,* and *CXCR4*) (Fig. 2).

### 3.3 Construction and validation of the autophagy prognostic signature based on the expression of significant DEARGs

**Figure 3:**
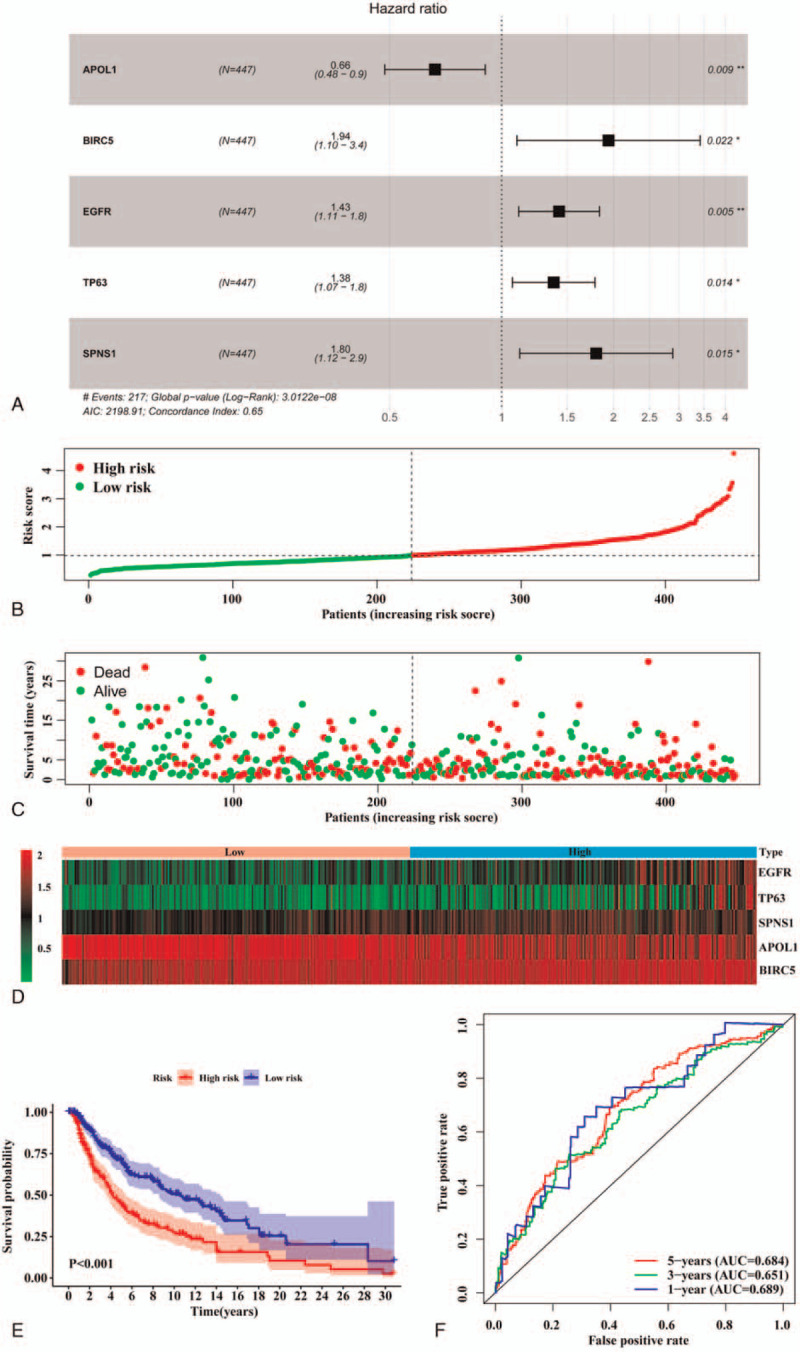
Construction of autophagy prognostic signature. (A) Autophagy prognostic signature was constructed through the multivariate Cox regression analysis to predict the prognosis of SKCM. (B–D) From top to bottom: risk score plot, survival status scatter plot, and heatmap of 5 key prognostic related genes in high-risk and low-risk groups. (E) Kaplan-Meier curve of high-risk and low-risk groups. (F) The time-dependent ROC curve to validate the ability of the constructed autophagy prognostic signature to predict 1-, 3-, and 5-year survival. AUC = area under the curve, ROC = receiver operating characteristic, SKCM = skin cutaneous melanoma.

Univariate Cox regression showed that 7 DEARGs are significantly associated with the prognosis of SKCM (Table 1). Further, multivariate Cox regression analysis was performed according to the univariate Cox regression results, and the results showed that 5 DEARGs (*APOL1, BIRC5, EGFR, TP63,* and *SPNS1*) are highly correlated with the prognosis of SKCM (Fig. 3A). These 5 DEARGs were selected to construct an autophagy prognostic signature (Fig. 3B–D). Kaplan-Meier curve indicted a significant difference between high-risk and low-risk groups with a *P* value from the log-rank test of <.001 (Fig. 3E). Area under the curves (AUCs) of the time-dependent ROC curves were 0.689, 0.651, and 0.684 for 1-, 3-, and 5-year survival, respectively (Fig. 3F).

**Table 1:** Univariate Cox regression analysis of DEARGs.

Gene	HR	95% CI	*P* value
APOL1	0.554	0.418–0.734	<.001
BIRC5	2.096	1.203–3.652	.009
EGFR	1.540	1.230–1.928	<.001
TP63	1.444	1.136–1.835	.003
ATG9B	1.774	1.338–2.353	<.001
SPNS1	1.931	1.265–2.948	.002
CXCR4	0.623	0.131–0.900	.012

### 3.4 Immunohistochemical analysis

**Figure 4:**
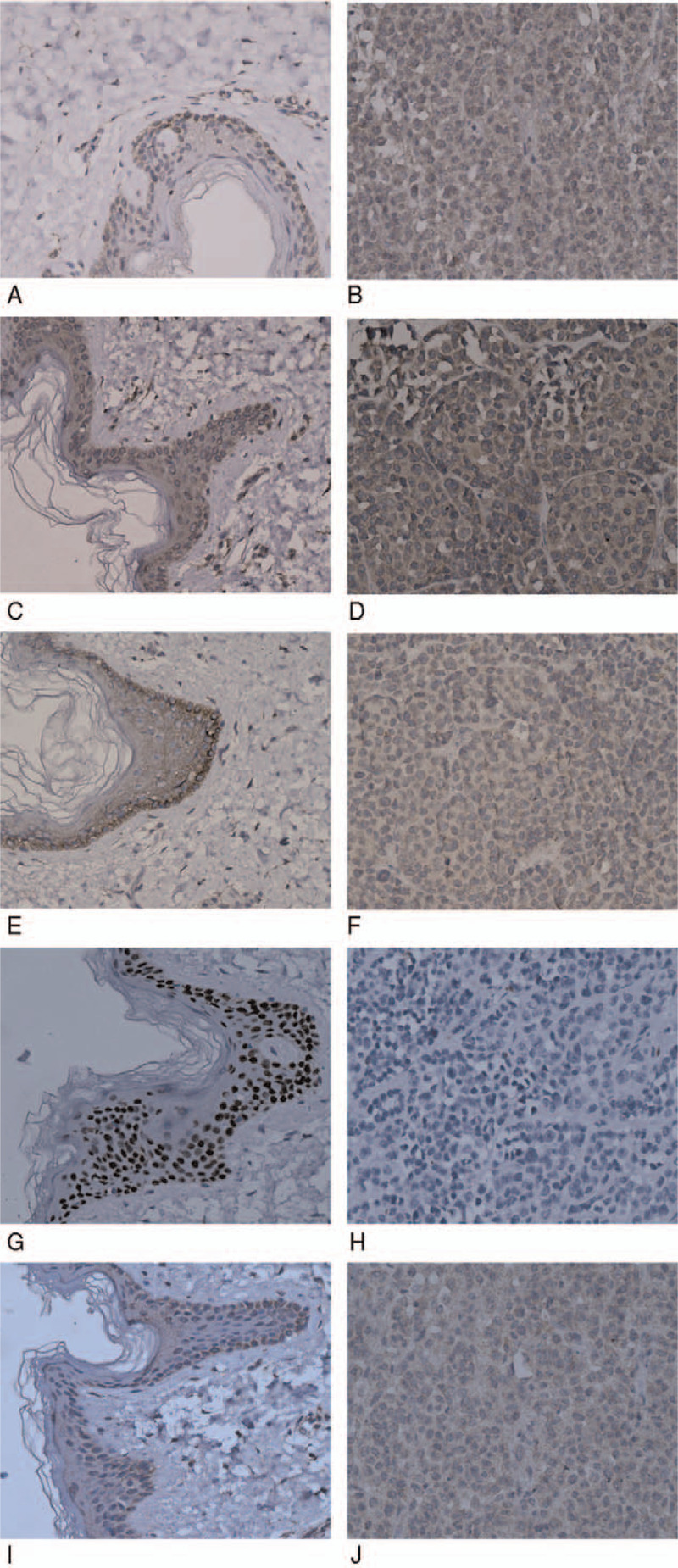
IHC staining of *APOL1, SURVIVIN, EFGR, TP63,* and *SPNS1* in SKCM and normal skin tissue (400×). Expression of *APOL1* (A), *BIRC5* (C), *EFGR* (E), *TP63* (G), and *SPNS*1 (I) in normal skin tissues. Expression of *APOL1* (B), *BIRC5* (D), *EFGR* (F), *TP63* (H), and *SPNS1* (J) in SKCM tissues. IHC = immunohistochemical. Images are at 40 × 10 magnification.

The expression of APOL1 and BIRC5 in all the SKCM specimen was found to be higher than in the normal skin specimens of all 10 tissue samples. However, the expression of EGFR, TP63, and SPNS1 in the SKCM specimens was found to be lower than in the normal skin specimens of all 10 tissue samples. The results of IHC analysis were consistent with those of DEARG identification (Fig. 4).

### 3.5 Autophagy prognostic signature as an independent prognostic factor

**Figure 5:**
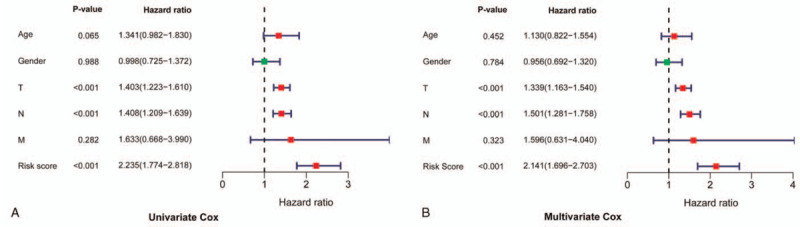
Univariate and multivariate Cox regression analyses assessed the independent prognostic value of autophagy prognostic signature in patients with SKCM. (A) Univariate Cox regression analysis. (B) Multivariate Cox regression analysis. SKCM = skin cutaneous melanoma.

To determine whether the risk score of the autophagy prognostic signature is an independent factor affecting the prognosis of SKCM, univariate and multivariate Cox regression analyses were performed. The results showed that T stage (*P* < .001, HR = 1.339, 95% CI = 1.163–1.540), N stage (*P* < .001, HR = 1.501, 95% CI = 1.281–1.758), and the risk score (*P* < .001, HR = 2.141, 95% CI = 1.696–2.703) are the independent prognostic factors for OS (Fig. 5 and Table 2). The high-risk group was significantly associated with poor prognosis in SKCM patients.

**Table 2:** Univariate and multivariate Cox regression analysis assessed the independent prognostic value of autophagy prognostic signature in SKCM patients.

	Univariate Cox			Multivariate Cox		
Covariates	HR	95% CI	*P* value	HR	95% CI	*P* value
Age	1.341	0.982–1.830	.065	1.130	0.822–1.554	.452
Gender	0.998	0.725–1.372	.988	0.956	0.692–1.320	.784
T	1.403	1.223–1.610	<.001	1.339	1.163–1.540	<.001
N	1.408	1.209–1.639	<.001	1.501	1.281–1.758	<.001
M	1.633	0.668–3.990	.282	1.596	0.631–4.040	.323
Risk score	2.235	1.774–2.818	<.001	2.141	1.696–2.703	<.001

### 3.6 Construction and validation of a predictive nomogram

**Figure 6:**
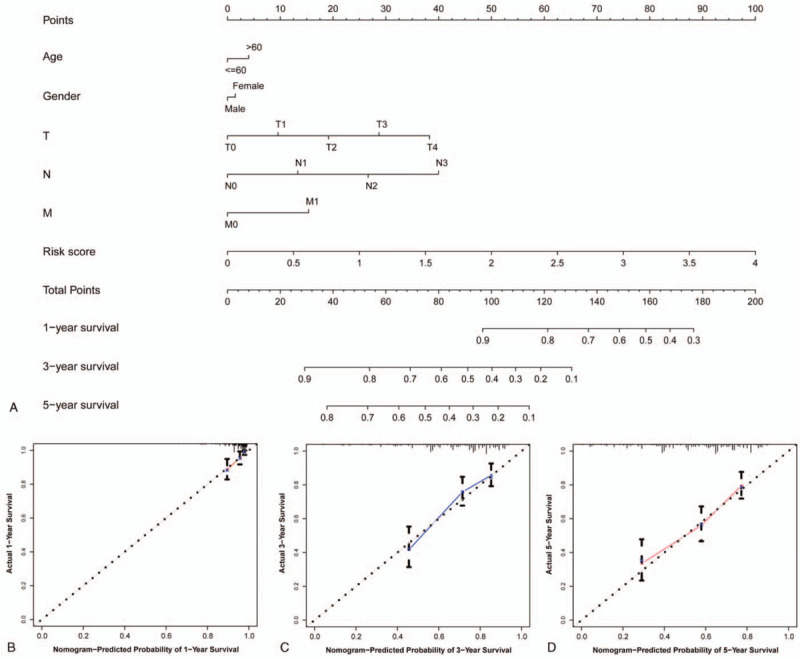
Construction and validation of a predictive nomogram. (A) A nomogram was constructed to predict 1-, 3-, and 5-year events (mortalities). (B–D) The calibration plot for internal validation of the nomogram.

A comprehensive nomogram was constructed on the basis of the risk score and traditional clinical characteristics to predict OS in SKCM patients. The results confirmed that the risk score has a particularly large impact on the prognosis of SKCM. The impact of other clinical characteristics on risk points was relatively small (Fig. 6A). Calibration plots of 1-, 3-, and 5-year OS of SKCM were in complete agreement between nomogram predictions and actual observations (Fig. 6B–D).

### 3.7 High- and low-risk groups showed different phenotypes

**Figure 7:**
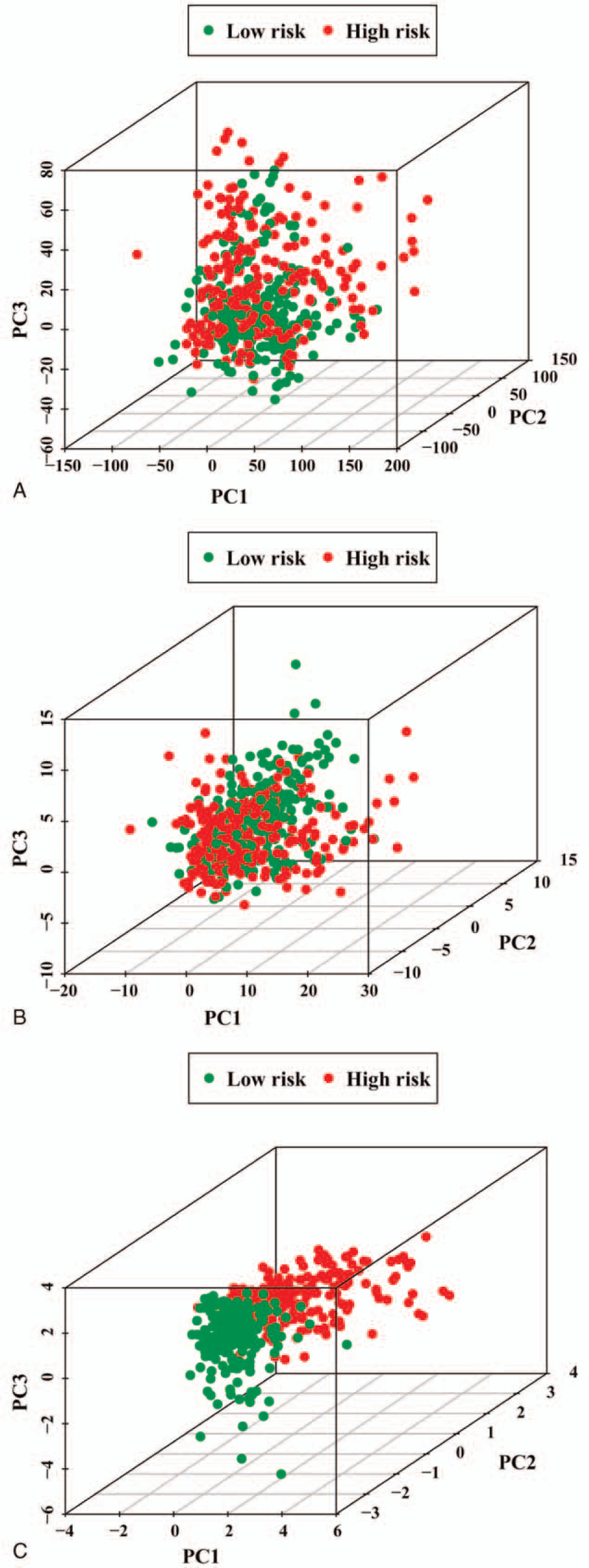
PCA between high-risk and low-risk groups based on different classification methods. (A) All genes. (B) ARGs. (C) Risk genes. PCA = principal components analysis.

Based on expression profiles of all the genes, ARGs and risk-related genes, PCA was performed to investigate differences between high-risk and low-risk populations (Fig. 7A–C). Our results indicated that patients in the high-risk or low-risk groups are distributed in discrete directions, suggesting that they represented different phenotypes. Therefore, according to the autophagy prognostic signature, patients in the high-risk group can be distinguished from those in the low-risk group.

### 3.8 GSEA

**Figure 8:**
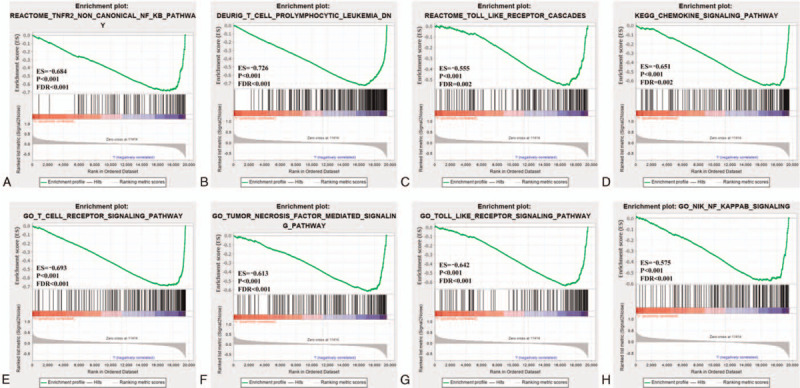
GSEA enrichment between the high-risk and low-risk groups. GSEA results for the (A–D) c2-reference and (E–H) c5-reference gene sets for the low-risk group. ES = enrichment score, FDR = false discovery rate, GSEA = gene set enrichment analysis.

Results of GSEA indicated that many differences exist in C2 and C5 gene sets in the low-risk group. It was mainly enriched in the toll-like receptor, chemokine, T cell receptor, PI3K-Akt, and MAPK signalling pathways (Fig. 8).

## 4 Discussion

Autophagy plays a vital role in the occurrence of many types of tumours and cancer progression. Moreover, the mechanisms are too complicated, and most of them are still unknown. Inhibition of autophagy was reported to reduce focal adhesions’ degradation, thereby reducing tumour cells’ motility. Autophagy plays a vital role in tumour cell movement and invasion in vivo and in vitro.^[12]^ Although many studies have confirmed that autophagy plays a vital role in cancer and that autophagy is closely related to the occurrence and malignant process of SKCM, its molecular mechanism is complicated. Hence, this study mainly focused on revealing potential molecular mechanisms of autophagy related to the prognosis of SKCM. In the current study, we found that 5 ARGs are significantly related to the prognosis of SKCM, and the signature established by these 5 ARGs can effectively predict the prognosis of SKCM patients. The 5 ARGs were: *APOL1, BIRC5, EGFR, TP63*, and *SPNS1*.

The PI3K-Akt signalling pathway has now become an important target for the cancer treatment.^[13]^ Dysregulation of the EGFR is known to be closely related to the malignant transformation and progression of many cancers. EGFR regulates many processes, such as cell proliferation and growth. The combination of EGFR and homologous ligands can autophosphorylate receptor tyrosine kinases and further activate the downstream PI3K-Akt pathway. The signalling pathway activates downstream corresponding effector molecules to regulate multiple cellular physiological processes. It is essential for cell proliferation, growth, survival, movement, and metabolism.^[14]^ Excessive activation of this pathway exists in human malignant tumours and is related to the development of cancer. Therefore, one of the important methods to treat tumours is to use molecular targets in the PI3K-Akt signalling pathway for rational drug designing.^[14]^ Cyclin D1 mainly regulates the transition of the cell cycle from the G1 to S phase. In many cancers, cyclin D1 is one of the downstream components of the PI3K-Akt signalling pathway. In SKCM, the upregulation of *Lyn* can promote the expression of cyclin D1 by activating the PI3k-Akt signalling pathway to promote the proliferation of SKCM cells.^[15]^ Therefore, we speculate that EGFR increases the expression of cyclin D1 by activating the PI3K-Akt signalling pathway to promote the proliferation of SKCM cells.

The activation of the MAPK signalling pathway plays a vital role in the biology of many cancers. It is involved in the transduction of extracellular signals such as growth factors and hormones to the nucleus, which ultimately leads to cell proliferation, differentiation, and survival.^[16–18]^ EGFR is one of the upstream effector molecules of the MAPK pathway, and its expression level plays a vital role in the MAPK signalling pathway.^[19]^ The expression level of EGFR can be increased by promoting phosphorylation, thereby activating the MAPK pathway. On the contrary, the expression level of EGFR can also be reduced by inhibiting phosphorylation to inhibit the MAPK pathway.^[20]^ The activation of the EGFR/MAPK pathway can promote the occurrence and metastasis of various cancers, such as hepatocellular carcinoma and oesophageal cancer.^[21,22]^

Similar to many tumours, abnormal activation of the MAPK signalling pathway is common in SKCM. Abnormal activation of the MAPK signalling pathway leads to SKCM cell cycle dysregulation and inhibition of cell apoptosis, which is a core step in the progression of SKCM. Abnormal MAPK pathway activation can be observed in 90% of SKCM cells.^[23,24]^ Therefore, the activation of EGFR/MAPK promotes the proliferation and metastasis of SKCM.

In summary, EGFR can simultaneously activate the PI3K-Akt and MAPK signalling pathways to promote SKCM cells proliferation. Jakob et al^[25]^ reported that mutations in codons 12, 13, or 61 of NRAS lead to the prolongation of the state of NRAS active GTP binding, thereby maintaining the NRAS signalling and physiologically involving in SKCM proliferation and survival through the MAPK and PI3K pathways.^[25,26]^ Therefore, we speculate that EGFR causes the progression of SKCM by activating the PI3K-Akt and MAPK signalling pathways.

APOL1 is an important signal protein that plays a vital role in regulating cell homeostasis, death, and survival. APOL1 can activate and transport LC3-II to maintain intracellular phospholipid homeostasis.^[27]^ Disruption of these functions may lead to a series of diseases including cancer. APOL1 has protective effect on normal renal cells, Inhibiting the expression of APOL1 can promote the progression of renal cell carcinoma.^[28]^

BIRC5 is at the crossroads of many cancer cell signalling networks and has been confirmed to be related to the progression of various malignant tumours.^[29–31]^ Zu et al^[32]^ reported that zinc fingers of the proto-oncogene and the BTB domain containing 7A can significantly upregulate the expression of BIRC5 to promote the development of breast cancer. OCT4 can upregulate the expression of BIRC5 and CCND1 by enhancing its promoter activity, these factors jointly promote the proliferation of liver cancer cells and aggravate prognosis of patients with Hepatocellular Carcinoma (HCC). The co-inhibition of OCT4 and BIRC5 may be beneficial to the treatment of HCC.^[33]^ Additionally, the high expression of BIRC5 promotes the proliferation of tumour cells in various tumour tissues, such as renal cell carcinoma, non-small cell lung cancer, and cervix cancer, and is associated with a worse prognosis.^[34–36]^

Several studies have reported that TP63 is involved in tumorigenesis through multiple mechanisms. The expression of TP63 was increased in approximately 25% of squamous cell carcinomas in multiple organs, including the lung, head, neck, and oesophagus.^[37]^ A study indicated that p63 is highly expressed in skin basal cell carcinoma.^[38]^ In addition, a high incidence of TP63 gene mutations in SKCM samples (14.7% of the samples) was reported.^[39]^ Matin et al^[40]^ reported that as many as 60% of SKCM p63-positive specimens (63/121) are positively correlated with the poor prognosis of SKCM.

Regarding *SPNS1*, only a few studies have been conducted; however, its relationship with tumours has not been reported. Nakano^[41]^ reported that the defect in *SPNS1* causes abnormal lysosomal function in zebrafish and short life span. In our study, the expression level of *SPNS1* in patients with SKCM in the relatively low-risk and high-risk groups was significantly increased, which was significantly correlated with poor prognosis. Further in-depth research is needed to confirm and clarify the association between Spns1 and SKCM.

However, this study has certain limitations. First, we downloaded the clinical data of SKCM through the data set of the UCSC XENA database. Most of the clinical data on this website are from medical institutions in European and American countries. Therefore, the accuracy of conclusions and the model constructed in our research should be further ascertained while extending them to patients in other regions; clinical data from more patients in other regions should be collected for verification. Second, although we experimentally verified the expression of genes involved in the construction of the model in the sample and identified the molecular mechanism through bioinformatics methods, basic experiments are required to further verify these findings.

Despite these limitations, to the best of our knowledge, this study is the first to use bioinformatics and IHC analyses to reveal the relationship between ARGs and the prognosis of SKCM. In addition, GSEA was used to determine the potential molecular mechanisms underlying ARGs that may affect the prognosis of patients with SKCM. Follow-up experimental studies are warranted to verify these findings. The ARGs identified in this study may become new targets for the treatment of SKCM.

In summary, *APOL1, BIRC5, EGFR, TP63,* and *SPNS1* are the potential prognostic biomarkers for patients with early SKCM and participate in signalling pathways such as PI3K-Akt and MAPK to affect the occurrence and development of SKCM.

## 5 Conclusion

The current study identified a 5-ARG signature that could be a useful prognostic biomarker for the risk classification of patients with SKCM. ARGs may affect the prognosis in part by regulating the PI3K-Akt and MAPK signalling pathways. These findings provide novel insights into the relationship between ARGs and prognosis of SKCM and lay a foundation for improving survival of patients with SKCM.

## Acknowledgments

We are grateful to Dr. Xinli Zhan (Spine and Osteopathy Ward, The First Affiliated Hospital of Guangxi Medical University,) for his kindly assistance in all stages of the present study.

## Author contributions

**Conceptualization:** Shian Liao, Juliang He, Chong Liu, Zide Zhang.

**Data curation:** Shian Liao, Juliang He, Hongyu Liao, Zuowei Liao.

**Formal analysis:** Shian Liao, Juliang He, Chaojie Yu.

**Funding acquisition:** Shian Liao, Juliang He, Jian Guan, Hao Mo.

**Investigation:** Shian Liao, Juliang He, Zhenchao Yuan, Xinli Zhan.

**Methodology:** Shian Liao, Juliang He, Tuo Liang.

**Project administration:** Shian Liao, Juliang He.

**Resources:** Shian Liao, Juliang He, Zhaojun Lu.

**Software:** Shian Liao, Juliang He, Guoyong Xu.

**Supervision:** Shian Liao, Juliang He, Zequn Wang.

**Validation:** Shian Liao, Juliang He, Jiarui Chen, Xinli Zhan.

**Visualization:** Shian Liao, Juliang He, Jie Jiang.

**Writing – original draft:** Shian Liao, Juliang He, Xinli Zhan.

**Writing – review & editing:** Shian Liao, Juliang He, Xinli Zhan.
